# Effect of *Lactobacillus gasseri* PA3 on gut microbiota in an in vitro colonic simulation

**DOI:** 10.1002/fsn3.1236

**Published:** 2019-11-14

**Authors:** Shasha Xiang, Jian Fu, Kun Ye, Yiqing Zheng, Xuan Zhu, Jie Chen, Yuewen Chen

**Affiliations:** ^1^ School of Food Science and Bioengineering Zhejiang Gongshang University Hangzhou China; ^2^ Eurofins Technology Service Qingdao Co., Ltd Qingdao China

**Keywords:** colonic simulation, gut microbiota, *Lactobacillus gasseri* PA3, metabolisms, purines

## Abstract

It has been reported that *Lactobacillus gasseri* PA3 has an ability to absorb exogenous purines in the intestine to reduce a risk of gout and hyperuricemia. However, influences of this strain on gut microbiota and their metabolisms remain unclear. Herein, we aimed to investigate the effect of *L. gasseri* PA3 on microbiota composition and metabolisms. *L. gasseri* PA3 was isolated from yogurt and supplemented into a single‐stage colonic fermentation in a culture volume of 30 ml and subjected to in vitro colonic simulation for 8 days. Microbiota composition was determined with 16S rRNA (V3 + V4) sequencing, and their metabolisms were predicted by PICRUSt. Short‐chain fatty acids were measured by GC‐MS. We found that *L. gasseri* PA3 reduced the diversity of microbiota, increased the relative abundances of *Lactobacillus* (73.5%) and *Escherichia* (36.5%), and decreased *Bacterioides* and *Phascolarctobacterium*. Total amount of short‐chain fatty acids was found to decline. Fundamental metabolisms, especially nucleotide, was significantly higher after intervention with *L. gasseri* PA3, but the purine metabolism was lower, which means that PA3 might reduce uric acid concentrations by weakening purine metabolism. Our results indicated that *L. gasseri* PA3 can survive and play a role in the ascending colon environment. Therefore, the evaluation of the effect of *L. gasseri* PA3 on intestinal microbes and their metabolisms has great guiding significance for the development of treatment to prevent gout.

## Graphical abstract

We investigated the effect of *Lactobacillus gasseri* on microbiota in an in vitro colonic simulation system. Results showed that *L. gasseri* reduced the diversity of microbiota, increased the relative abundance of *Lactobacillus* (73.5%) and *Escherichia* (36.5%), and decreased *Bacterioides* and *Phascolarctobacterium*. Fundamental metabolisms, especially amino acid, were significantly reduced after intervention with *L. gasseri* PA3. The evaluation of the effect of *L*. *gasseri* PA3 on intestinal microbes and their metabolism has great guiding significance for the development of treatment to prevent gout.

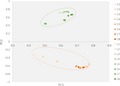

## 1 INTRODUCTION

Gut microbiota is an extremely dynamic and complicated ecological environment, which has numerous essential roles in human physiology and is related to chronic diseases like obesity, diabetes, and autism spectrum disorders (Kałużna‐Czaplińska, Gątarek, Chartrand, Dadar, & Bjorklund, 2017).More than 10^11^ cfu/g of microbes are found in the colon, which is their area of colonization (Dethlefsen, Huse, Sogin, & Relman, 2008). As the study of human or animal intestinal contents directly involves ethical issues and individual differences, in vitro simulated colonic fermentation becomes more and more popular, which is a feasible, fast, and widely used method to cultivate and explore gut microbiota (Williams et al., 2015).

*Lactobacillus* species, known as probiotics, are active microorganisms that act by maintaining the stability of the host intestinal microflora and producing beneficial metabolites for host, like promoting the ecological balance of the microbial flora, improving the structure of the intestinal flora, inhibiting the growth of harmful bacteria in the intestine, eliminating carcinogenic factors, enhancing immunity, and lowering cholesterol (Farnworth, 2008). Previous research works indicated that *Lactobacillus gasseri* PA3 has the ability to reduce purines, including inosine 5′‐monophosphate (IMP), inosine, hypoxanthine, guanosine 5′‐monophosphate (GMP), and guanosine in rats, but *L. gasseri* OLL2996 does not have the ability to absorb purines (Kano et al., 2018; Yamada et al., 2016, 2018, 2017). Further study shows that *L. gasseri* PA3 can lower serum uric acid levels even in patients with hyperuricemia and gout (Yamanaka, Taniguchi, Tsuboi, Kano, & Asami, 2018), which is caused by decrease of uric acid excretion or increase of uric acid accumulation because of abnormal purine metabolism (Richette & Bardin, 2010). In Japan, daily intake of dietary purines is recommended to be less than 400 mg to prevent gout and hyperuricemia (Kaneko, Aoyagi, Fukuuchi, Inazawa, & Yamaoka, 2014). Recent study of *L. gasseri* PA3 offers a new breakthrough in preventing gout, and not just relying on a controlled diet with less purine. However, the question of how PA3 will influence gut microbiota remains unclear.

PA3 enters the small intestine to weaken the absorption of exogenous purines. The arrival of new species will definitely affect other microorganisms and their metabolism. Irrespective of whether it can colonize the intestine or not, a new steady state of microbial community will be formed, to build a potential barrier against hyperuricemia and gout. This research on the change of intestinal microbial composition and metabolism induced by *L. gasseri* PA3 having an ability to adsorb purine has been undertaken, which can be a guideline for the development of an effective treatment for hyperuricemia and gout.

## 2 MATERIALS AND METHODS

### 2.1 Preparation of *Lactobacillus gasseri* PA3 supplements (LGS)

*Lactobacillus gasseri* PA3 with an ability of purine absorption was isolated from *Lactobacillus gasseri* PA3 drinking yogurt (Meiji) by the method of Jang et al. (2013) and cultured in Man Rogosa Sharpe (MRS) liquid medium at 37°C for 48 hr. MRS (g/L): peptone 10.0, beef extract 5.0, glucose 20.0, Tween 80 1.0, dipotassium phosphate 2.0, sodium acetate 5.0, triammonium citrate 2.0, magnesium sulfate 0.2, manganese sulfate 0.05, and agar powder 14.5. After 15 min of centrifugation at 6,000*g*, supernatant was discarded and isometric fermented nutritive medium (FNM) was added to suspend the cells. FNM (g/L) is composed of: peptone 3.0, corn starch 8.0, yeast extract 4.5, tryptone 3.0, Mucin 0.5, l‐cysteine hydrochloride 0.8, bile No.3 0.4, heme 0.05, sodium chloride 4.5, Tween 80 1.0, potassium chloride 2.5, potassium dihydrogen phosphate 0.4, magnesium chloride hexahydrate 4.5, calcium chloride hexahydrate 0.2, and 2 ml of trace elements stock solution (containing g/L: magnesium sulfate heptahydrate 3.0, ferrous sulfate heptahydrate 0.1, calcium chloride dihydrate 0.1, manganese chloride tetrahydrate 0.32, cobalt sulfate heptahydrate 0.18, copper sulfate pentahydrate 0.01, zinc sulfate heptahydrate 0.18, nickel chloride hexahydrate 0.092; Fan et al., 2016).

### 2.2 Fecal sample collection and pretreatment

Fecal samples were donated by six healthy adult women, aged 20–30 years, who did not have inflammatory bowel disease and irritable bowel syndrome, and did not receive antibiotics for at least 6 months. The above fecal samples were voluntarily provided by volunteers and informed consent was signed. Ten grams of fresh fecal samples were collected and microorganisms were fully suspended in 90 ml of sterile PBS (0.1 M, pH = 6.8) by stirring well. Then the suspension was filtered by a sterile gauze and made into microbial suspension. The whole operation was carried out in an anaerobic environment.

### 2.3 In vitro single‐stage colonic fermentation

**Figure 1:**
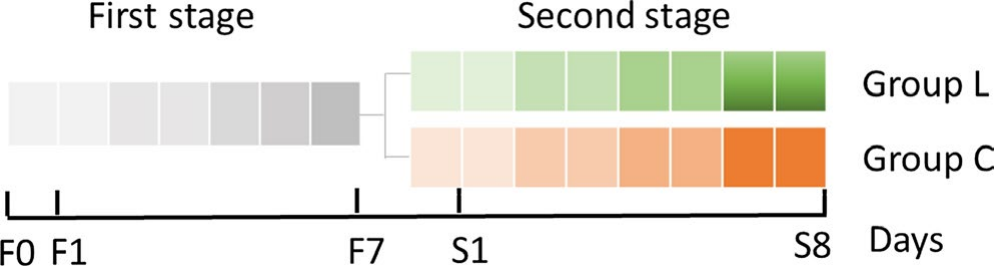
The schematic graph of in vitro single‐stage colonic simulation, each small square represents 1 day. First stage: stable period (F1–F7); second stage: *Lactobacillus gasseri* PA3 supplemental period (S1–S8, group L is supplemented with *L. gasseri* PA3 and group C with FMN as control group). Samples were collected and analyzed from day S1 to day S8

In vitro single‐stage colonic fermentation was conducted in Changdao Moni simulation system (CDMNS) at Zhejiang Gongshang University. About 10% of fecal microbiota (v/v) was inoculated into first‐stage (Figure 1) fermentation reactors with 600 ml FNM. The fermentation system approached stability after 7 days, and fermentation liquid was equally pumped into 2 reactors (second stage). One was supplied with 30 ml LGS (L) while the other was supplemented with an equivalent volume of FNM (control group [C]) on the first day of second‐stage process. During the entire fermentation process, the speed of stirring rotor was set at 120 r/min and maintained uniformly throughout the experiment. All the reactors were kept at 37°C, and 0.5 M NaOH and HCl were utilized to maintain pH values at 6.8. Nitrogen gas with a speed of 0.1 L/min was passed into every vessel for 30 min every morning, noon, and night to maintain an anaerobic environment. The consecutive fed‐batch fermentations were used by replacing nutrient medium at a flow speed of 37.5 ml/hr and were conducted for 24 hr per cycle. In the meantime, fermentation liquor was wasted out at a same velocity to balance the total amount of medium in the reactor. The samples of second stage were collected every 24 hr after supplementation for 8 days and kept at −20°C. The experiments were repeated in triplicate.

### 2.4 Short‐chain fatty acid assay

A volume of 0.5 ml of fermentation supernatant was taken from each sample and filtered through 0.22 μm filter membrane after centrifugation at 9570*g* for 2 min. The collected supernatant was again centrifuged at 10,000*g* for 5 min after thoroughly mixing with 10 ml of ethyl ether and 0.05 ml of sulfuric acid solution (50%). The mixture solution was investigated by gas chromatography–mass spectrometry (GC‐MS: 7890A‐5975C; Agilent) analysis with the following operating conditions: free fatty acid phase (FFAP) elastic quartz capillary column (30 m × 0.25 mm × 0.25 µm); carrier gas: 2 ml/min high purity nitrogen (≥99.999%); ion source type for mass spectrometer: electrospray ion source; ion source temperature: 350°C; interface temperature: 250°C; inlet temperature: 270°C; detector temperature (FID): 280°C; and injection volume: 2.0 μl.

### 2.5 DNA extraction, PCR amplification, and 16S rRNA gene analysis

DNA from samples of 2 reactors at second stage from days 1 to 8 was extracted using a MicroElute Genomic DNA Kit (D3096‐01; Omega, Inc.) according to the protocol. The total DNA was eluted in 50 µl of TE elution by a small modified version (the time of water bath to break the cell wall was extended to 0.5–1 hr) of the procedure described by the manufacturer (Qiagen). The final DNA concentration was determined by NanoDrop 2000 UV‐vis spectrophotometer (Thermo Scientific), and DNA quality was checked by 1% agarose gel electrophoresis. The V3–V4 hypervariable regions of the bacterial 16S rRNA gene were amplified with primers 319F (5′‐ACTCCTACGGGAGGCAGCAG‐3′) and 806R (5′‐GGACTACHVGGGTWTCTAAT‐3′) by thermocycler PCR system (GeneAmp 9700; ABI; Huang, Yang, & Li, 2016). The PCR reactions were conducted using the following program: 30 s of denaturation at 98°C, 35 cycles of 10 s at 98°C, 30 s for annealing at 54/52°C, 45 s for elongation at 72°C, and a final extension at 72°C for 10 min. PCR reactions were performed in triplicate using 20 μl of reaction mixture containing 4 μl of 5× FastPfu buffer, 2 μl of 2.5 mM dNTPs, 0.8 μl of each primer (5 μM), 0.4 μl of FastPfu polymerase, and 10 ng of template DNA. The resulting PCR products were extracted from the 2% agarose gel and further purified using the AxyPrep DNA Gel Extraction Kit (Axygen Biosciences) and quantified using QuantiFluor™ ‐ST (Promega) according to the manufacturer's protocol.

Purified amplicons were pooled in equimolar and paired‐end sequenced (2 × 300) on an Illumina MiSeq platform (Illumina) according to the standard protocols by LC‐Bio Technology Co. Ltd.16S rRNA gene sequences were processed as we previously described (Xu et al., 2018).

### 2.6 Statistics

All data are shown as the mean ± *SD*. The data were analyzed by variance and Duncan's test for multiple comparisons with SPSS ver. 17.0. A value of *p* < .05 was considered significant.

## 3 RESULTS

### 3.1 Effect of *Lactobacillus gasseri* PA3 on bacteria community

**Figure 2:**
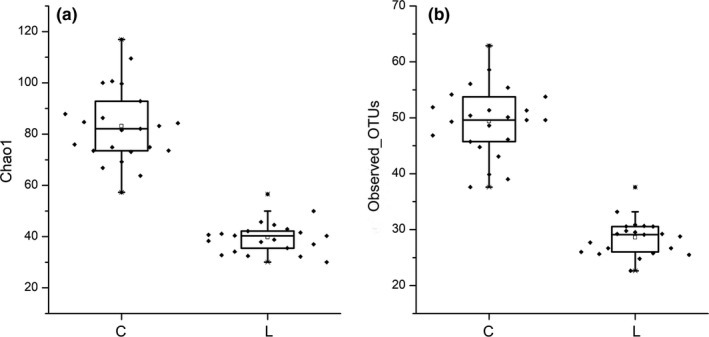
The Alpha diversity index, Chao1 (a), and observed_OTUs (b) of C (control) and L (*Lactobacillus gasseri*) group in vitro second‐stage colonic simulation

A total of 45 samples from two groups, with/without* L. gasseri* PA3, were chosen for microorganism profile analyses. After quality filtering, the 16S rRNA sequencing results produced 9,317,346 reads, giving a mean sample depth of 99,120 reads with a standard deviation of 79,078 reads. A total of 814 operational taxonomic units (OTUs) were obtained, and the relative abundance of OTUs showed the genera relative abundances in the samples. Chao1 and observed OTUs are two indices to indicate abundance and diversity of microbial community. As shown in Figure 2, diversities of the microorganisms in the L group were significantly lower than those in the C group.

**Figure 3:**
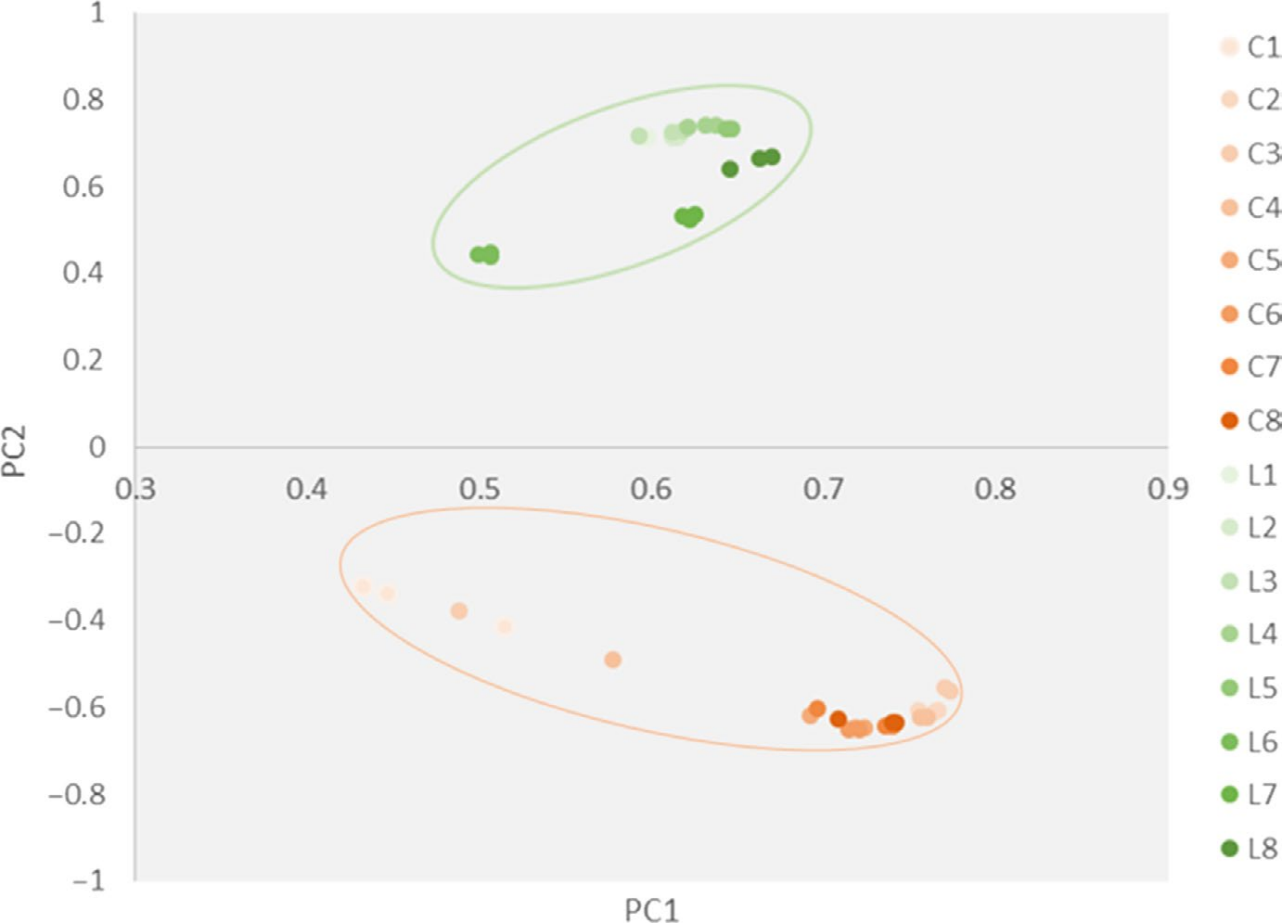
Principal components analysis of samples collected during 8 days of in vitro colonic simulation. The weight coefficients of PC1 and PC2 are 42.58% and 38.79%, respectively

**Figure 4:**
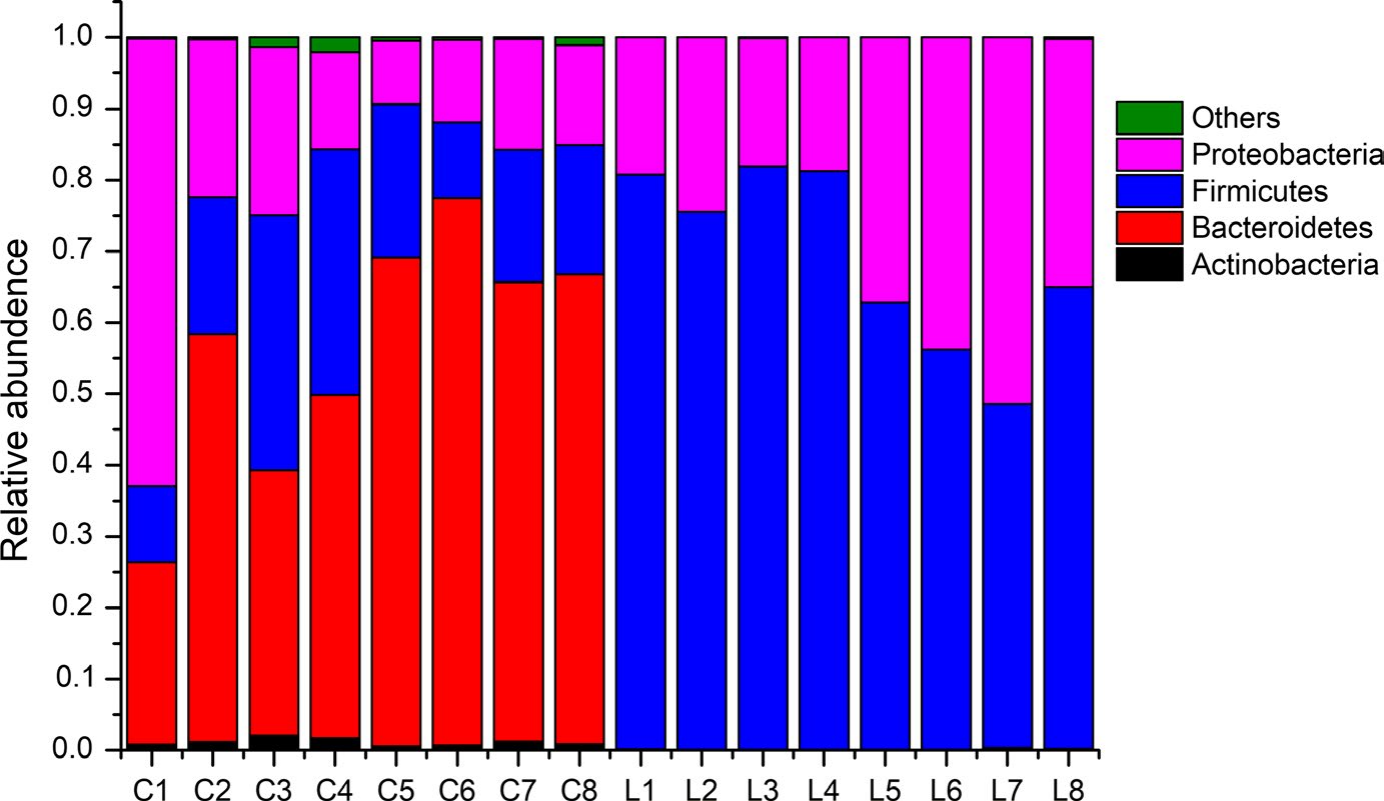
Effect of *Lactobacillus gasseri* on bacterial phylum alternation during the 8 days. C stands for control group, L stands for *L. gasseri* group, and figures stand for the day

**Figure 5:**
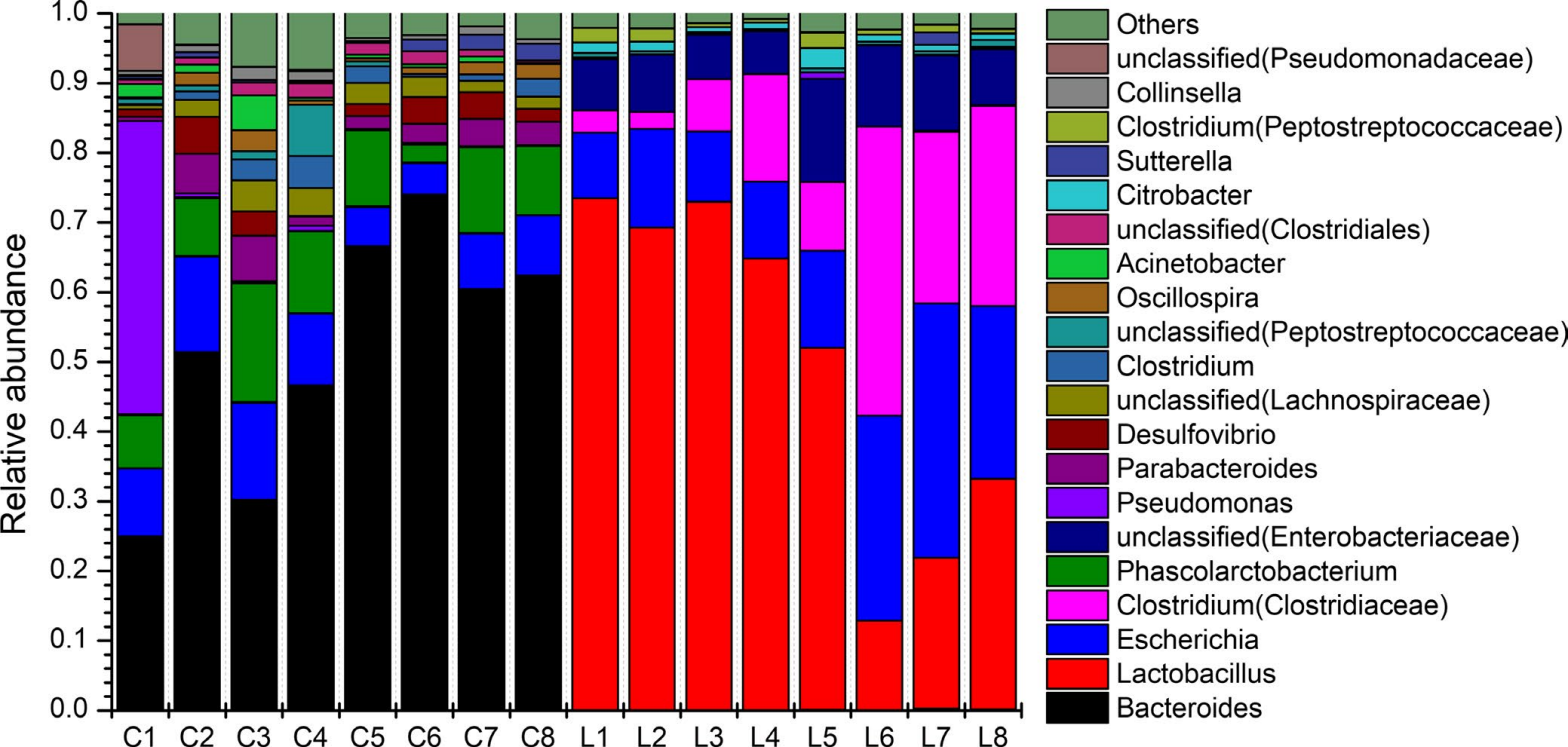
Effect of *Lactobacillus gasseri* on bacterial genera alternation during the 8 days. C stands for control group, L stands for *L. gasseri* group, and figures stand for the day

**Figure 6:**
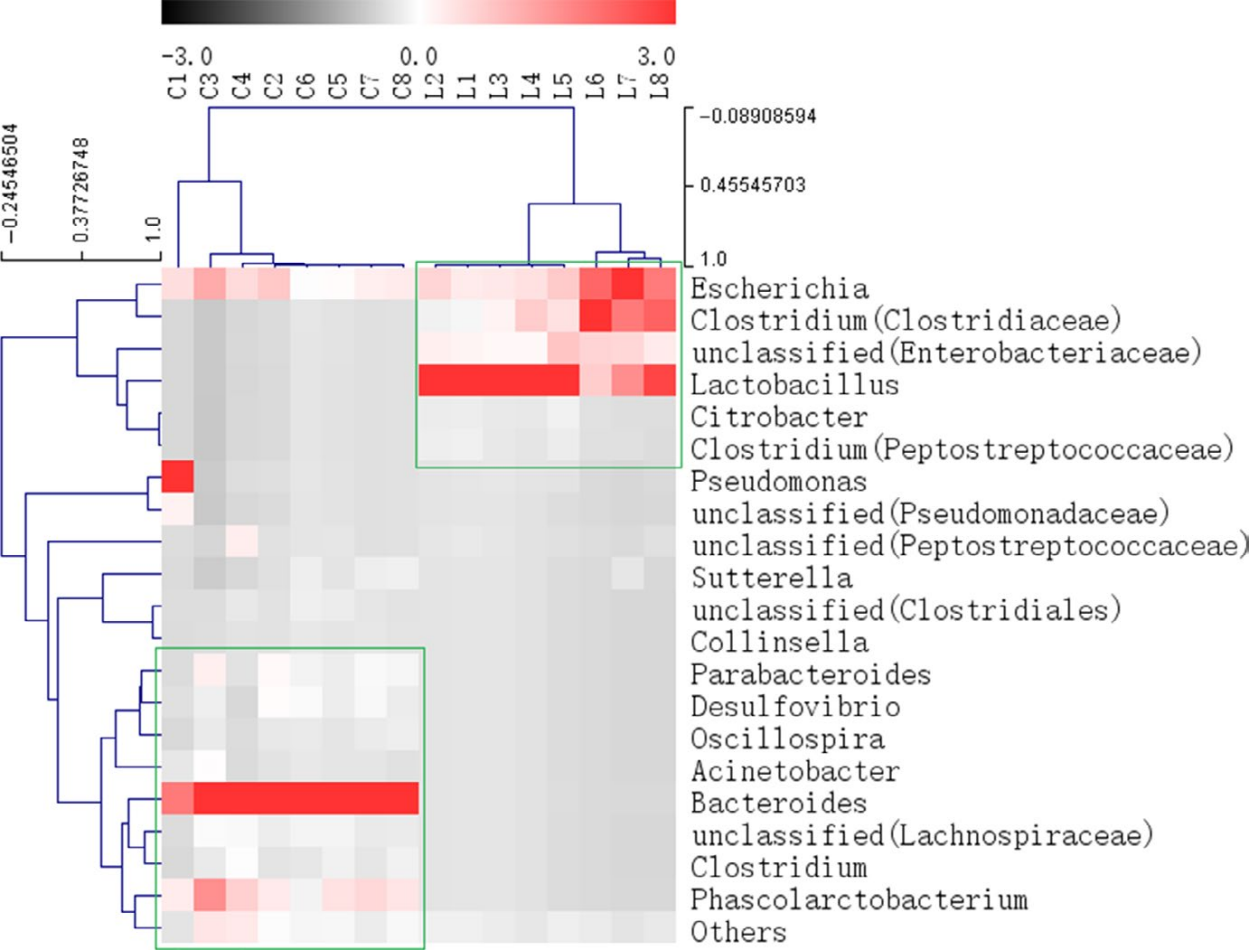
Heatmap and hierarchical clustering (Euclidean distance) on the taxonomic genus level. C stands for control group, L stands for *Lactobacillus gasseri* group and figures stand for the day

A principal component analysis (PCA) with a two‐dimensional principal component cumulative variance of 81.73% showed that the human intestinal microflora significantly changed under the influence of exogenous *L. gasseri* PA3 (Figure 3). The control group and *L. gasseri* group consisted of two different sections and were significantly distinguished from each other. With the addition of *L. gasseri* PA3, the composition of the intestinal microflora also had undergone great changes. At the level of the phylum, *Firmicutes* and *Proteobacteria* were the main groups in the *L. gasseri* groups, while *Bacteroidetes* was predominant in the control group, followed by *Firmicutes* and *Actinomycetes* (Figure 4). With the passage of time, the relative abundance of every phylum showed a parabolic shape. The content of *Proteobacteria* in the *L. gasseri* group was increased from the beginning to the end of the experiment. The content of *Firmicutes* in the *L. gasseri* group was gradually reduced. The relative amount of *Bacteroidetes* in the control group gradually increased, while that of *Proteobacteria* was significantly decreased. At the genus level (Figure 5), *L. gasseri* PA3 increased the relative abundances of *Lactobacillus* (73.5%) and *Escherichia* (36.5%), and decreased *Bacterioides* and *Phascolarctobacterium*; the predominant group in the *L. gasseri* group was *Lactobacillus*, but it was gradually replaced by *Escherichia*. On the sixth day, the relative abundance of *Lactobacillus* was recovered, but the decreasing trend of *Escherichia* was not observed. The concentration of predominant bacterial genus in the control group, *Bacteroides,* was relatively increased over time. According to the heatmap cluster analysis (Figure 6), the difference between the *L. gasseri* group and the control group was obvious. The pattern of inter‐group differences was similar to that shown in the PCA result.

### 3.2 Effect of *Lactobacillus gasseri* PA3 on short‐chain fatty acids

**Figure 7:**
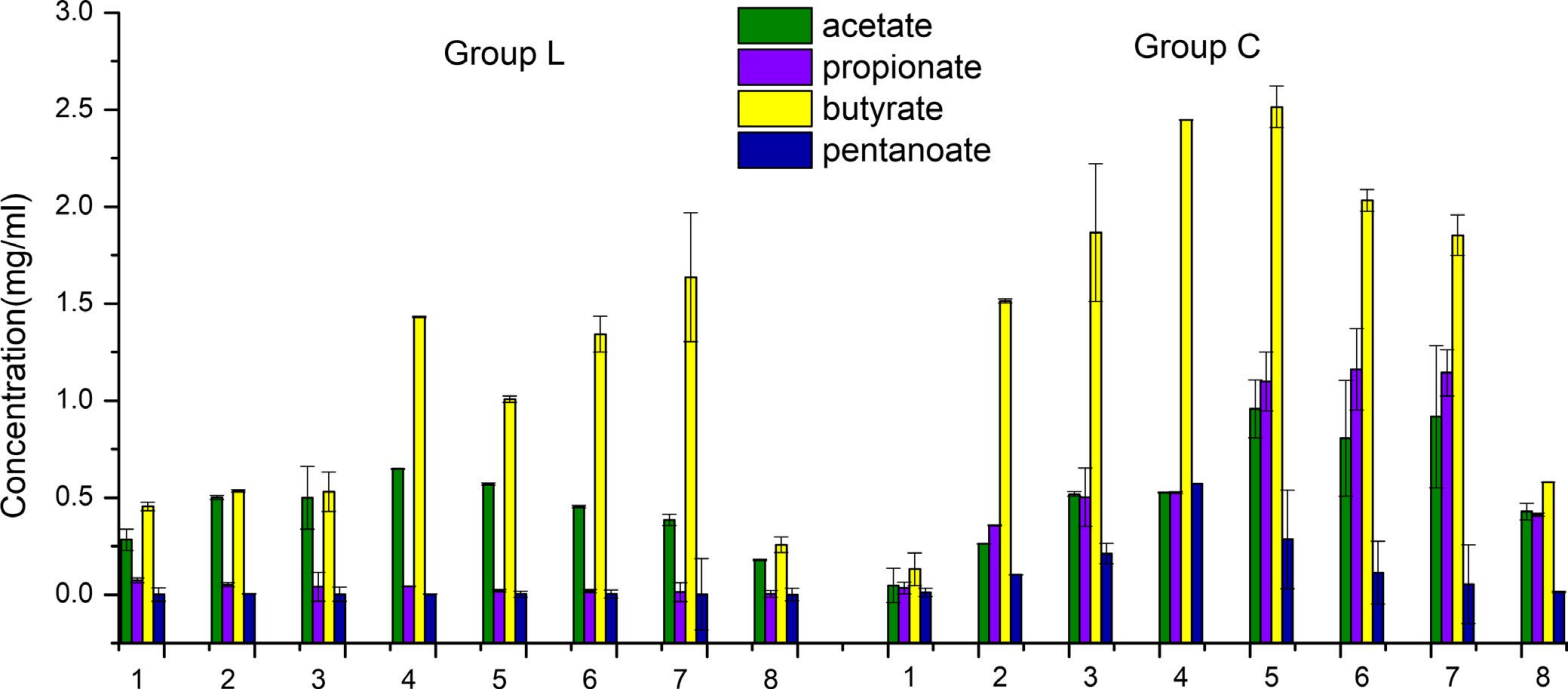
Effect of *Lactobacillus* *gasseri* on short‐chain fatty acids during the 8‐day simulation period (mg/ml)

The total concentration of short‐chain fatty acids in the *L. gasseri* group was lower than that in the control group, but both groups showed a parabolic shape of short‐chain fatty acid (SCFA) content. The concentration of propionate and pentanoate in the *L. gasseri* group was almost difficult to detect. The concentration of acetate and butyrate was low, but their proportion was higher. The level of acetate in the *L. gasseri* group was lower in the second‐stage fermentation than in the control group, and the concentration of acetate in the *L. gasseri* group reached the peak on the fourth day. During the first 6 days, butyrate concentration in the *L. gasseri* group was lower than that in the control group, and a reverse trend was observed in the last two days, which reached the peak on the seventh day. Acetate and butyrate showed a negative correlation between each other (Figure 7).

### 3.3 Relationship between samples, microbiota, and SCFAs via canonical correspondence analysis

**Figure 8:**
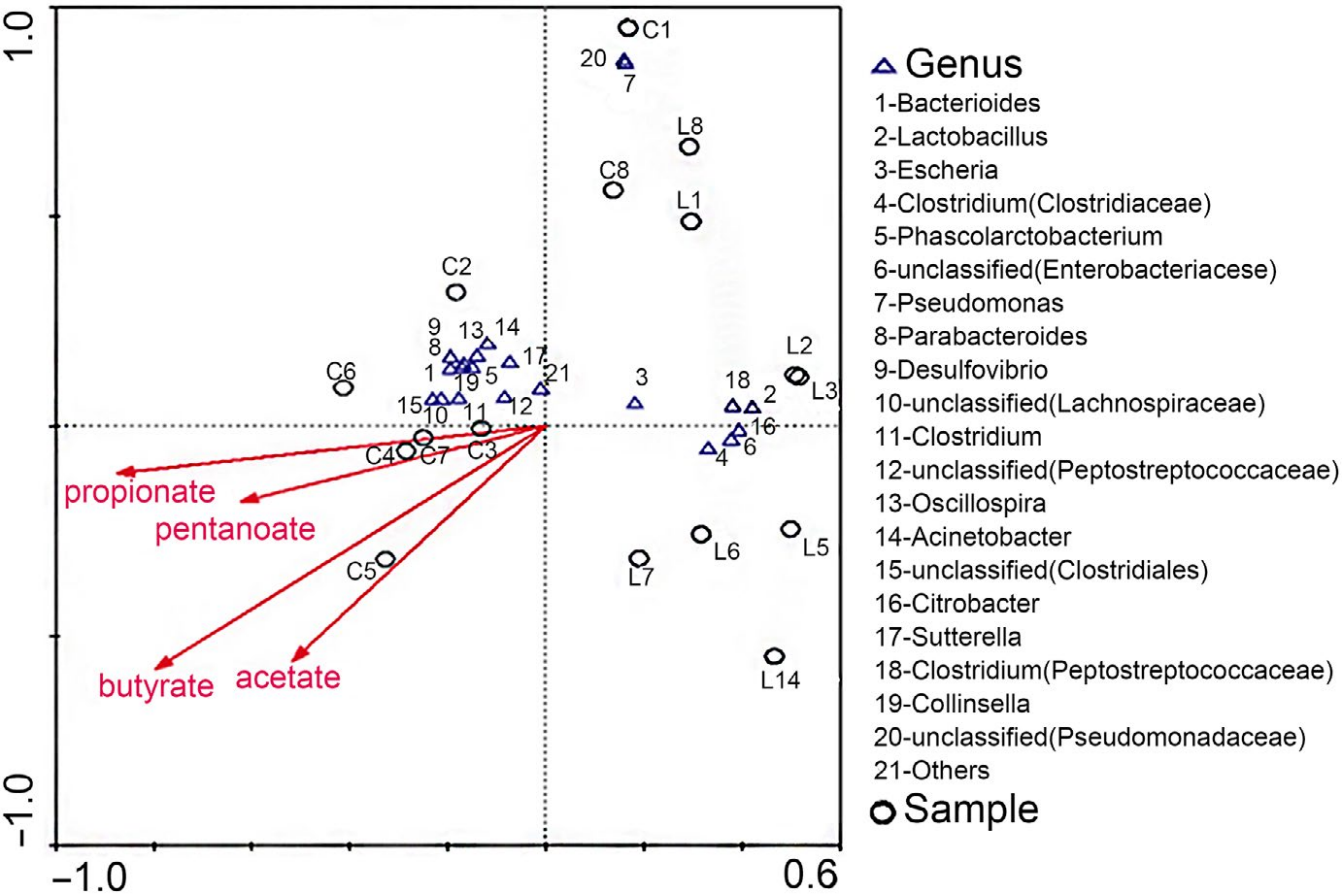
Canonical correspondence analysis. Cycles stand for samples, triangles stand for genus, and arrows stand for different short‐chain fatty acids. The closer the two points, the higher was the similarity of a community structure of two samples. Arrows suggested distinct factors of influences. When an angle between influencing factors (or between factors and samples) was an acute angle, these two factors were positively correlated. On the contrary, an obtuse angle had a negative correlation. The longer the ray, the heavier was the effects of factors

We found that nearly half of the bacteria are positively related to the metabolism of acetate and butyrate, while most of the bacteria are extensively related to propionate and pentanoate. Here, we also found that *Escherichia*, *Lactobacillus*, *Clostridium*, and *Citrobacter* had a negative correlation with SCFAs in this simulation. Samples of *L. gasseri* group were mainly concentrated in the lower right region. Due to the rich species diversity, the control group and most of the microorganisms mainly fell in the upper left block. The samples collected on the first and the last day of the two groups were similarly located in the upper right block (Figure 8).

### 3.4 Metabolism and function prediction of gut microbial community

**Figure 9:**
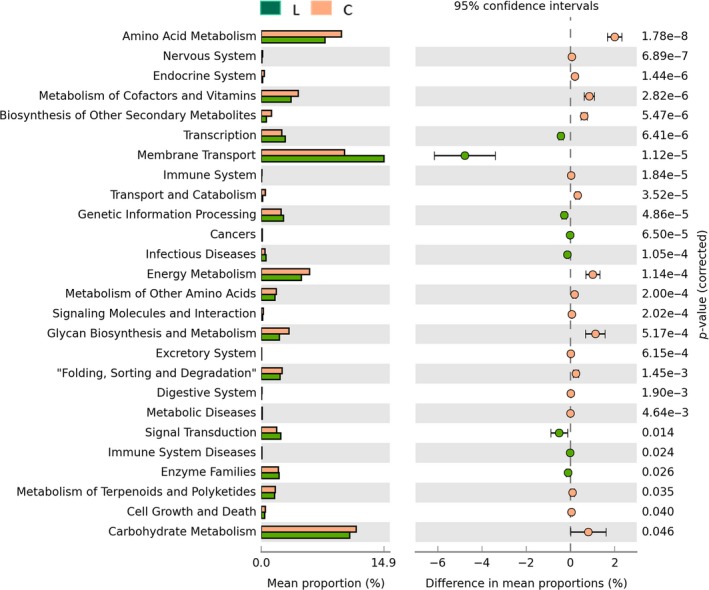
Comparisons of the predominant gene pathways of the bacterial microbiota in different groups as predicted by PICRUSt. Green stands for *Lactobacillus* *gasseri* group and brown stands for control group

**Figure 10:**
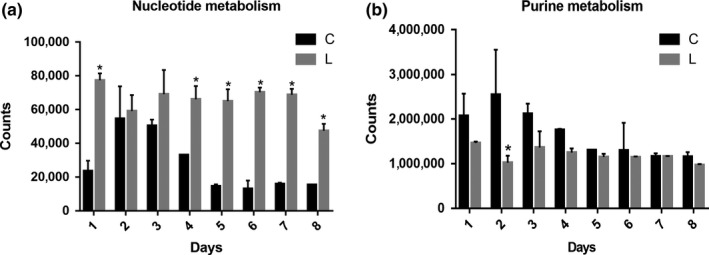
Nucleotide metabolism (a) and purine metabolism (b) of tertiary metabolic pathways of Kyoto Encyclopedia of Genes and Genome as predicted by PICRUSt

16S rRNA marker gene sequences were used to predict functional profiling of microbial communities, 41 secondary metabolic pathways, and 327 tertiary metabolic pathways of Kyoto Encyclopedia of Genes and Genomes (KEGG), including nucleotide metabolism and purine metabolism, which were obtained through phylogenetic investigation of communities by reconstruction of unobserved states (PICRUSt) prediction (Table S1). After eight days of metabolism prediction, the metabolism of L group was lower than that of group C. Twenty‐six secondary pathways show significant differences between the two groups. The most significant observation was that the amino acid metabolism in *L. gasseri* group was reduced. Moreover, important pathways related to energy metabolism, carbohydrate metabolism, and metabolism of cofactors and vitamins were all reduced in contrast to control group, while membrane transport increased (Figure 9). The results of tertiary metabolic pathways show that nucleotide metabolism is stronger in group L. Although nucleotide metabolites such as purines are very threatening to gout, the purine metabolism pathway, which is the production pathway of uric acid, in the group L was weaker than that in the group C, especially on the second day (Figure 10).

## 4 DISCUSSION

In this study, in vitro colonic simulation of *L. gasseri* PA3 digestion showed that dietary intake of *Lactobacillus* led to a significant change of intestinal microbe composition and that *Lactobacillus* became dominant bacteria at later stages, thus decreasing microbial diversity. Due to specificities of individuals, gut microbiota varies widely and showed a high degree of dynamism, but intestinal microbiota of individuals consisted of *Firmicutes* and *Proteobacteria*, which occupy 80%–90% of the relative abundance of the intestinal flora (Park, Omura, Fujita, Sato, & Tsunoda, 2017). However, overdoses of *L. gasseri* PA3 caused the irreversible decrease of *Bacteroides*. In the late fermentation stage, the relative abundance of *Proteobacteria* was gradually increased by nearly 50%, and *Lactobacillus* gradually decreased, indicating that *Lactobacillus* and *Escherichia* had a certain competitive relationship. We also observed that *Lactobacillus* was recovered on the sixth day, and *Clostridium* and *Escherichia* were reduced. This rebound phenomenon observed in the relative abundance of *Lactobacillus* may indicate that *Lactobacillus* began to adapt to the competition in intestine and started to reproduce. However, previous studies by Prilassnig, Wenisch, Daxboeck, and Feierl (2007) found that *L. gasseri* could not be detected in feces after one week, after probiotics were administered to healthy individuals. In this study, we found that *L. gasseri* can survive and reproduce in the ascending colon portion.

Dietary fiber, proteins, and peptides are metabolized by the microbiota of the cecum and colon after escaping from host enzyme digestion. The main products of microbial fermentation activity in the gut are SCFAs (Macfarlane & Macfarlane, 2012). SCFAs can directly activate G‐coupled receptors, inhibit histone deacetylases, and act as an energy source for small intestinal epithelial cells. Therefore, they affect various physiological processes and may lead to health and diseases (Koh, De Vadder, Kovatcheva‐Datchary, & Bäckhed, 2016). Butyrate is the energy source for small intestinal epithelial cells, and it can also exhibit anti‐inflammatory effects by blocking NF‐κB activation, which is considered a potential substitute for antibiotics (Canfora, Jocken, & Blaak, 2015). Acetate is the major metabolite of most bacterial glycolytic pathways in the intestine and is the first short‐chain fatty acid found in the intestinal tract of newborns. An in vitro study of the effect of physiological concentrations of short‐chain fatty acids on intestinal mucosal barrier in neonatal rats showed that physiological concentrations of acetic acid are more effective in maintaining normal intestinal mucosal barrier function than those of butyrate, and that acetate can be metabolized by various intestinal microbes, including *Bifidobacteria* (Li et al., 2015). Although the relative abundance of *Bifidobacteria* in this study did not belong to top 20 genera, there was a significant difference between two groups (*p* < .01), indicating more acetate was produced from *Bifidobacteria* in the control group. Propionate absorbed by the colon is mainly metabolized by the liver, participates in gluconeogenesis, and inhibits cholesterol synthesis (Backhed et al., 2004). Propionic acid is mainly produced by *Bacteroidetes* and *Firmicutes* via the succinic acid metabolic pathway (Yamanaka et al., 2018). The detection level of propionic acid in *L. gasseri* group was very low, due to dysregulated propionate metabolism caused by decreasing of *Bacteroides*. Acetate mostly is produced by Wood–Ljiungdahl pathway of *Clostridium* and *Streptococcus* or from pyruvate via acetyl‐CoA of *Akkermansia muciniphila*,* Bacterioides*,* Bifidobacterium*, and *Prevotella*. Butyrate is produced by acetate CoA‐transferase route of *Anaerostipes, Coprococcus,* and *Eubacterium* (Koh et al., 2016). The large amount of *Lactobacillus* may weaken the formation of acetate while it might strengthen the production of lactate.

Metabolic and functional predictions of gut microbiota showed that the amino acid metabolism of the gut microbiota was significantly decreased in the gut environment where *L. gasseri* PA3 was abundantly present. Nucleotide metabolism was stronger in the group L which was characterized by tertiary metabolic pathways. Although nucleotide metabolites such as purines are much threatening to gout, the purine metabolism pathway, which is the production pathway of uric acid, was weaker in the group L than in the group C, especially on the second day.

We propose that *L. gasseri* PA3 exerted an effect by reducing uric acid concentration by weakening the metabolism of purines, which may be similar to the mechanism of previous report which demonstrated a decrease in the uric acid production by interfering with purine metabolic pathway (Yang & Yuan, 2013). This mechanism is different in *Lactobacillus bulgaricus* which first absorbs uric acid and then degrades it (Bai, Jiang, & Jiang, 2014). However, weather purines in the intestine can be reduced like a mechanism reported by Yamada et al. (2017, 2018) remains unclear and needs further study.

*Lactobacillus gasseri* PA3 can reduce purines of intestine in rats and lower serum uric acid levels in patients.The pathway further validates the role of *L. gasseri* PA3 in preventing and relieving gout, which is of great significance for the prevention and treatment of hypertension, hyperlipidemia, heart disease, diabetes, and cancer (Farnworth, 2008; Koh et al., 2016). In this study, *L. gasseri* PA3 can survive and play a role in the ascending colon environment. Therefore, the evaluation of the effect of *L*. *gasseri* PA3 on intestinal microbes and their metabolisms has great guiding significance for the development of treatment to prevent gout.

The evaluation of probiotics is often done through the combination of external model, animal test, and clinical trial. Although in vitro fermentation model is fast and economical, it is widely used to assess the potential of probiotics in large quantities of foods that contain probiotics, and to ensure the effectiveness of the animal and clinical research.

## CONFLICT OF INTEREST

The authors declare that they do not have any conflict of interest.

## ETHICAL APPROVAL

We complied with the US Federal Policy for the Protection of Human Subjects. All the study protocols and procedures were ethically reviewed and approved by The Ethical Committee of Zhejiang Gongshang University.

## INFORMED CONSENT

Informed consent was obtained from all the volunteers who donated fecal samples.
